# Detailed T1-Weighted Profiles from the Human Cortex Measured in Vivo at 3 Tesla MRI

**DOI:** 10.1007/s12021-018-9356-2

**Published:** 2018-01-19

**Authors:** Bart Ferguson, Natalia Petridou, Alessio Fracasso, Martijn P. van den Heuvel, Rachel M. Brouwer, Hilleke E. Hulshoff Pol, René S. Kahn, René C.W. Mandl

**Affiliations:** 1 Brain Center Rudolf Magnus, Department of Psychiatry, Brain Division, University Medical Center Utrecht, Utrecht University, HPNR A01.126, Heidelberglaan 100, 3584 CG Utrecht, The Netherlands; 2 Radiology Department, Imaging Division, University Medical Center Utrecht, Heidelberglaan 100, 3584 CG Utrecht, The Netherlands; 3 Experimental Psychology, Helmholtz Institute, Utrecht University, Heidelberglaan 1, 3584 CS Utrecht, The Netherlands; 4 CNSR, Psykiatrisk Center Glostrup, Ndr. Ringvej 29-67, DK-2600 Copenhagen, Glostrup Denmark

**Keywords:** 3 Tesla, 7 Tesla, Automatic segmentation, Laminar structure, Cortical profiles, Myeloarchitectonics

## Abstract

Studies into cortical thickness in psychiatric diseases based on T1-weighted MRI frequently report on aberrations in the cerebral cortex. Due to limitations in image resolution for studies conducted at conventional MRI field strengths (e.g. 3 Tesla (T)) this information cannot be used to establish which of the cortical layers may be implicated. Here we propose a new analysis method that computes one high-resolution average cortical profile per brain region extracting myeloarchitectural information from T1-weighted MRI scans that are routinely acquired at a conventional field strength. To assess this new method, we acquired standard T1-weighted scans at 3 T and compared them with state-of-the-art ultra-high resolution T1-weighted scans optimised for intracortical myelin contrast acquired at 7 T. Average cortical profiles were computed for seven different brain regions. Besides a qualitative comparison between the 3 T scans, 7 T scans, and results from literature, we tested if the results from dynamic time warping-based clustering are similar for the cortical profiles computed from 7 T and 3 T data. In addition, we quantitatively compared cortical profiles computed for V1, V2 and V7 for both 7 T and 3 T data using a priori information on their relative myelin concentration. Although qualitative comparisons show that at an individual level average profiles computed for 7 T have more pronounced features than 3 T profiles the results from the quantitative analyses suggest that average cortical profiles computed from T1-weighted scans acquired at 3 T indeed contain myeloarchitectural information similar to profiles computed from the scans acquired at 7 T. The proposed method therefore provides a step forward to study cortical myeloarchitecture in vivo at conventional magnetic field strength both in health and disease.

**Electronic supplementary material:**

The online version of this article (10.1007/s12021-018-9356-2) contains supplementary material, which is available to authorized users.

## Introduction

The cerebral cortex has a distinct laminar structure and the different layers are associated with different neuronal functions. To better understand how neurological and psychiatric diseases affect the brain it is not only important to determine which of the cortical brain regions are implicated but also to determine which of the layers may be involved in these regions (Charney et al. 2013). The layers of the cortex can be defined on the basis of the size, shape, and arrangement of neuronal cell bodies, known as cytoarchitecture (Brodmann 1909; von Economo and Koskinas 1925) or by the presence of myelinated nerve fibers, referred to as myeloarchitecture (Vogt and Vogt 1903). The cerebral cortex can be parcellated into regions with the same cyto- or myeloarchitecture (Nieuwenhuys 2013). Studying the cytoarchitecture, however, usually requires invasive staining methods and can therefore only be applied post-mortem in humans (Zilles and Amunts 2015). In contrast, the myeloarchitecture can be non-invasively studied in vivo using magnetic resonance imaging (MRI), rendering MRI well-suited to study large groups of patients and/or healthy subjects. This is particularly important when studying psychiatric diseases, where the enrolment of large groups is necessary because of the expected small effect sizes associated with this type of research.

Different types of MRI contrasts can be used to study the myeloarchitecture including T1 contrast, for which the signal variation in grey matter is for a large part explained by myelin concentration (Eickhoff et al. 2005; Lorio et al. 2016; Stüber et al. 2014). Other possible contrasts that can reveal laminar structure include T2*-weighted magnitude or phase images, which are putatively sensitive to iron or ferritin (Duyn et al. 2007; Duyn and Schenck 2016; Fukunaga et al. 2010)**.** Much effort has been put into high-field high-resolution MRI myelin mapping, especially in the occipital and primary visual area (Abdollahi et al. 2014; Clark et al. 1992; Sereno et al. 2013; Trampel et al. 2011), the auditory cortex (Wasserthal et al. 2014) and sensorimotor cortex (Weiss et al. 2011), and recently, whole brain (Fracasso et al. 2016). In addition, sensorimotor (Sanchez-Panchuelo et al. 2012; Sánchez-Panchuelo et al. 2014) and auditory (De Martino et al. 2015; Dick et al. 2012) cortical regions have been investigated with functional MRI, also in relation to local myelination (Lutti et al. 2014). However, scanning large groups of subjects using ultra-high field MRI is not straightforward because of the limited availability of ultra-high field scanners as well as the additional financial costs involved.

A large body of studies that used MRI conducted at conventional field strengths (e.g. 3 T) revealed cortical thinning in, for instance, schizophrenia (Van Haren et al. 2011; Wheeler et al. 2014; Xiao et al. 2015), depression (Tu et al. 2012) and bipolar disorder (Elvsashagen et al. 2013; Lan et al. 2014). Although the resolution of scans that were used to measure cortical thickness was considered fine enough to compute the inner (white matter) and outer (pial) boundary of the cortex, it was too coarse to obtain information on individual layers, leaving the question whether this thinning was due to a mechanism that affects all cortical layers of a particular region similarly, or that specific cortical layers were more affected than others. The highest resolution that can be achieved for these standard T1-weighted (T1-w) MRI scans is primarily determined by the available scanning time and the static magnetic field strength on which the scanner operates and is typically in the order of 0.8–1.0 mm isotropic for 3 Tesla (T). A number of studies have used MRI at conventional field strength to obtain information on the laminar structure. These studies, however, required special types of MRI scans, with long acquisition times (Glasser and Van Essen 2011; Mangeat et al. 2015), only focussed on a particular part of the brain (Bridge et al. 2005; Walters et al. 2003), or did not directly image the layers but only inferred the involvement of certain layers by comparing the thickness of adjacent gyri and sulci in a particular brain region (Wagstyl et al. 2015).

Here we propose a new fully automatic post-processing method to extract information on myeloarchitecture from a T1-w scan that is routinely acquired at 3 T. This method (described in detail in the “Methods” section) combines deconvolution and profile realignment to compute one detailed average T1-w based signal profile per brain region, assuming equal laminar organization within that region of interest.

We start out with a basic simulation to demonstrate that application of this method to a simulated T1-w volume results in a detailed average profile that contains information on signal characteristics and to assess the sensitivity of the method to potential artefacts such as deconvolution-related Gibbs ringing.

For further assessment of the method we acquired whole brain T1-w scans from three healthy volunteers that were scanned both at a 3 T and a 7 T scanner. For 3 T a standard T1-w acquisition protocol (3T-T1) was used while for 7 T a novel whole-brain, high-resolution (0.5 mm isotropic) T1-w MPRAGE sequence (MS7T) was used that is adapted to be sensitive to myelin content within grey matter (Fracasso et al. 2016). This scan was previously validated with histology (Fracasso et al. 2016) and therefore considered apt to validate the profiles computed from the standard 3 T T1-w scans.

In particular, the primary visual cortex (V1) is used to assess (MRI) acquisitions and methods that focus on myeloarchitecture (Hopf 1955, 1956, 1957; Waehnert et al. 2016). The reason for this is that in V1, layer 4 (containing the myelinated band called the outer line of Baillarger and in V1 also known as the stria of Gennari; see Supplementary Fig. S1) is heavily myelinated and even visible to the naked eye. The signal from V1 is often contrasted to that of the secondary visual cortex (V2) which lies adjacent to V1 but has a different laminar organization. Besides comparing V1 with V2, we included V7, which is a (occipital) visual area as well but has a lower myelin content compared to V1 and V2 (Glasser and Van Essen 2011). Four primary motor and sensorimotor regions were also included in this study. Three of these regions (BA1, BA2, BA3b) lie within the primary somatosensory cortex and one region (BA4) is part of the motor cortex.

In a first quantitative analysis we use hierarchical crisp clustering based on dynamic time warping to determine if profiles computed in 3 T data cluster in a way similar to the profiles computed in 7 T data. In a second quantitative analysis we compare the profiles computed for the visual areas using prior knowledge. Here we expect that the signal measured in the middle of the profile is the highest for V1 because of the line of Gennari in V1, followed by the signal measured at the same location for V2. For V7 we expect that the signal is relatively lowest. If the clustering patterns are significantly similar (first analysis) and the relative signal differences are significant (second analysis) for 7 T profiles as well as 3 T profiles (and with the same ordering) then we consider this as an indication that 3 T profiles indeed bear information similar to 7 T profiles. In a third analysis we assess the possible influence of acquisition-related Gibb’s ringing.

## Methods

### Outline of the Method

**Fig. 1:**
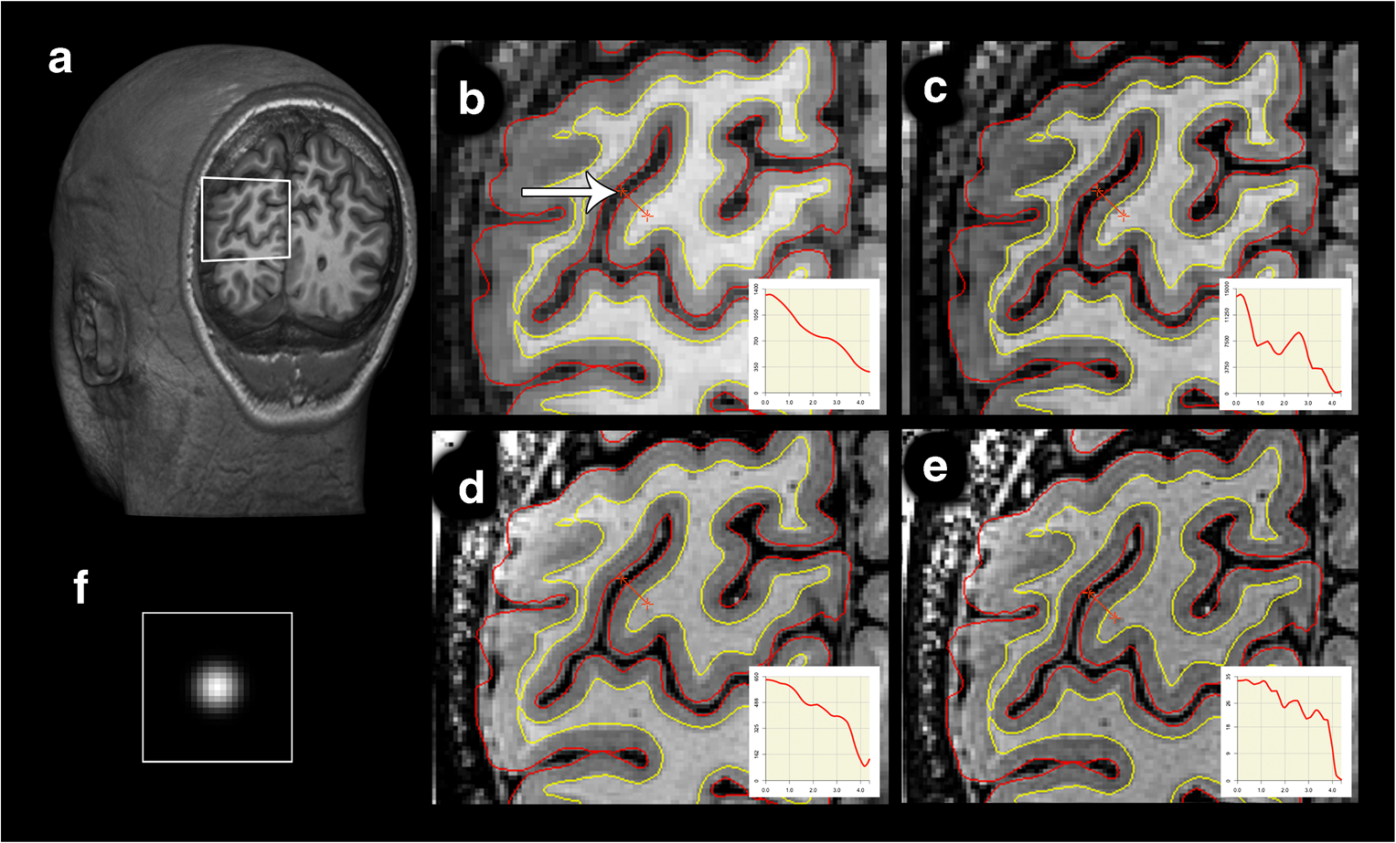
Result of deconvolution of 3 T and 7 T scans, coronal slice. Effect of deconvolution shown in a coronal section of the occipital lobe for one participant for the area of interest displayed in the white rectangle in (**a**). The four panels on the right indicate the area of interest in an original (**a**) and deconvoluted (**b**) 3 T T1w 0.8 mm isotropic scan and in an original (**d**) and deconvoluted (**e**) 7 T myelin-sensitive T1w 0.5 mm isotropic scan. Freesurfer white (red) and pial (yellow) surfaces are overlaid. Insets display signal intensity sampled along displayed orange ruler (marked by the white arrow), with 3DSlicer 4.4.0, which is 4.2 mm in length and runs from white matter to CSF. Please note that this sample is drawn in a 2D projection and is not necessarily perpendicular to the cortical mantle. (**f**) depicts the PSF used

The inner (white to grey matter) and outer (pial) boundaries of the cortex are computed from the T1-w scan acquired at 3 T using FreeSurfer (Fischl 2012). These boundaries are then superimposed on an upsampled and 3D–deconvoluted version of the original 3 T scan (see Fig. 1) after which a set of signal profiles is computed for each cortical region. The deconvolution step increases the detail in extracted profiles at the expense of a reduction in signal to noise ratio (SNR). To compensate for possible misalignment between profiles, the set of profiles are aligned using parametric time warping (ptw) (Eilers 2004; Gerretzen et al. 2015) and then averaged (increasing SNR) resulting in one average profile. With ptw, one profile is selected as the best representative for the set of profiles, and all other profiles are then shifted and scaled linearly towards this best representative profile. A major advantage of ptw is that it is computation friendly both in terms of memory usage and computation time (Eilers 2004), and is therefore well-suited to align large sets of profiles. Additional bootstrapping is applied over the alignment and averaging procedure to both increase stability and to be able to assess stability of the average profile. These steps are explained in more detail, below. The software is currently under development but the code used in this study is available on request.

### Deconvolution

Deconvolution is a mathematical operation that removes ‘blurring’ effects and was performed as follows. First, the resolution of the original volume (either 3T-T1 or MS7T) was doubled in all three directions using nearest neighbour interpolation. Next, 3D deconvolution was applied using the Parallel Iterative 3D Deconvolution toolbox (Wendykier 2009) (implemented in ImageJ / FIJI) with the following settings: Wiener Filter Preconditioned Landweber method; 1 iteration; 3D Gaussian-shaped point spread function (PSF) with a full-width at half maximum of 5 voxels in a volume with size 25x25x25 voxels (Fig. 1f); anti-ringing step on; divergence detection on; gamma = 0. The anti-ringing step reduces ringing at the boundaries of the volume.

### Cortical Profile Sampling

Cortical profiles for a selected brain region were sampled using a combination of Freesurfer and in-house developed software that was written in R (R Core Team 2014), and in C using the minc software library (http://www.bic.mni.mcgill.ca). Freesurfer label files were created using the thresholded Brodmann Area (BA) map for the regions BA1, BA2, BA3b, BA4a, V1 and V2 (Desikan et al. 2006; Fischl et al. 2004, 2008). The label for V7 was created using the PALS B12 Visuotopic atlas (https://surfer.nmr.mgh.harvard.edu/fswiki/PALS_B12). These label files were used to create pairs of coordinates on the inner and outer boundary of the cortex (linkage nodes, see (Lerch and Evans 2005)). Thus, a signal profile is here defined as the T1 signal measured along a straight line running from a point on the inner boundary to the corresponding point on the outer boundary (where the correspondence by the points is created by the expansion of the outer surface from the inner surface). Information on local cortical curvature was retrieved from the standard Freesurfer output. Cortical thickness was calculated based on the Euclidian distance between inner and outer boundary coordinate pairs. Intensity values were sampled at 100 equidistant points along a profile running from the inner to the outer boundary using linear interpolation. The sampling of data points was extended in both directions of the profiles with 30 points in each direction to allow for small shifts and scaling during the alignment of the set of profiles.

### Profile Alignment

As previously argued (Koopmans et al. 2011), simply averaging over a set of profiles results in a loss of detail. This may be due to small errors made during boundary extraction (e.g. white to gray matter boundary), for example due to suboptimal scan quality (e.g. subject motion), which could lead to suboptimal placement of inner and outer boundaries and thereby to translations and scaling deviances of profiles. Another reason may be the fact that differences in cortical curvature induce differences in relative thickness of the individual layers. That is, the superficial layers are thinner in gyri compared to the deeper layers (and vice versa for the sulci) (Waehnert et al. 2014).

To correct for the effects of the former — the translation and scaling deviances — we developed a procedure based on parametric time warping (ptw) (Eilers 2004) using the ‘ptw’ package implemented in R (Gerretzen et al. 2015). The effects of the latter can be mitigated by averaging only over profiles that were computed for brain areas with a similar cortical curvature. We therefore included only profiles for a region for which the corresponding cortical curvature was within ±1 standard deviation from the mean cortical curvature of that region. Further homogenization is obtained by only including profiles from parts of the region with a similar cortical thickness. Here, ± 0.5 standard deviation from the mean thickness was used as selection criterion. We note that alternative methods to correct for local cortical curvature exist and may be used in future implementations, for instance methods based on the heat conduction equation (Annese et al. 2004, 2005), a laplacian equation (De Martino et al. 2013) or the equi-volume model (Waehnert et al. 2014).

To prevent loss of detail in the computed average cortical profile, a highly accurate alignment of the set of profiles is required. Here the computation of the alignment transformations using parametric time warping was not performed directly on the profile data but on a version of the profiles from which the contribution of the slowly varying signal baseline was removed. This was done to ensure that the alignment is based on local changes in signal intensities that may represent laminar information rather than the less informative slowly varying baseline of the profiles. For this, we modelled the baseline using a cubic spline with the degrees of freedom set to 7 and subtracted the baseline from the original signal resulting in the version of the profile used to compute the transformations.

To align a complete set of profiles, first, a single profile was selected for which the weighted cross correlation is highest with all other profiles (a standard function in the ptw package). This profile was then regarded as the best representative profile of the set of profiles and was subsequently used as target profile to which all other profiles were aligned. Shift and linear scale transformation values were calculated using ptw with weighted cross correlation (triangle width parameter set to 20) as optimisation metric (Bloemberg et al. 2010; Meerts et al. 2004). The parameter search space was not restricted. The alignment parameters were applied to the original cortical profiles, and missing values, created at the beginning or end of a profile that has been shifted inward, were replaced with the respectively first or last value of the original profile. After alignment, the individual profiles were averaged, resulting in one average cortical profile.

### Bootstrapping

One concern may be that of model shine-through, meaning that the shape of the average profile is largely determined by the shape of the target profile. Although such model shine-through would not render the method invalid (as the choice of the target profile is purely data-driven) it may reduce stability as the choice of the target profile becomes crucial. To increase stability of the computed average profile, bootstrapping was applied over the alignment and averaging step. A total of 500 bootstrap samples were created. For each bootstrap sample, a random set of profiles (with the same number of profiles as the original set) was drawn with replacement from the original set of profiles. This sample was then aligned to the best reference profile selected from this sample and averaged, yielding an average profile. Finally, computing the mean over the 500 average profiles resulted in one bootstrap analysis mean (BAM) profile.

An additional advantage of the bootstrap procedure is that is it can be used to obtain information on the level of stability of features that reflect the level of detail along the BAM profile. One could select salient features, compute them for each bootstrap sample average profile and then determine their distributions. For example, local maxima (‘peaks’) and minima (‘valleys’) detected for each bootstrap sample average profile can serve as salient features. These peaks and valleys are computed from a smoothed version of the input (cubic spline, degrees of freedom set to 15) to remove possible noise-related minima and maxima, and are defined as the positions where the second derivative equals zero. The distribution of the peaks and valleys computed over the 500 bootstrap sample average profiles can then be used to assess the stability of the peaks and valleys in the corresponding BAM profile.

### Simulation Experiment

**Fig. 2:**
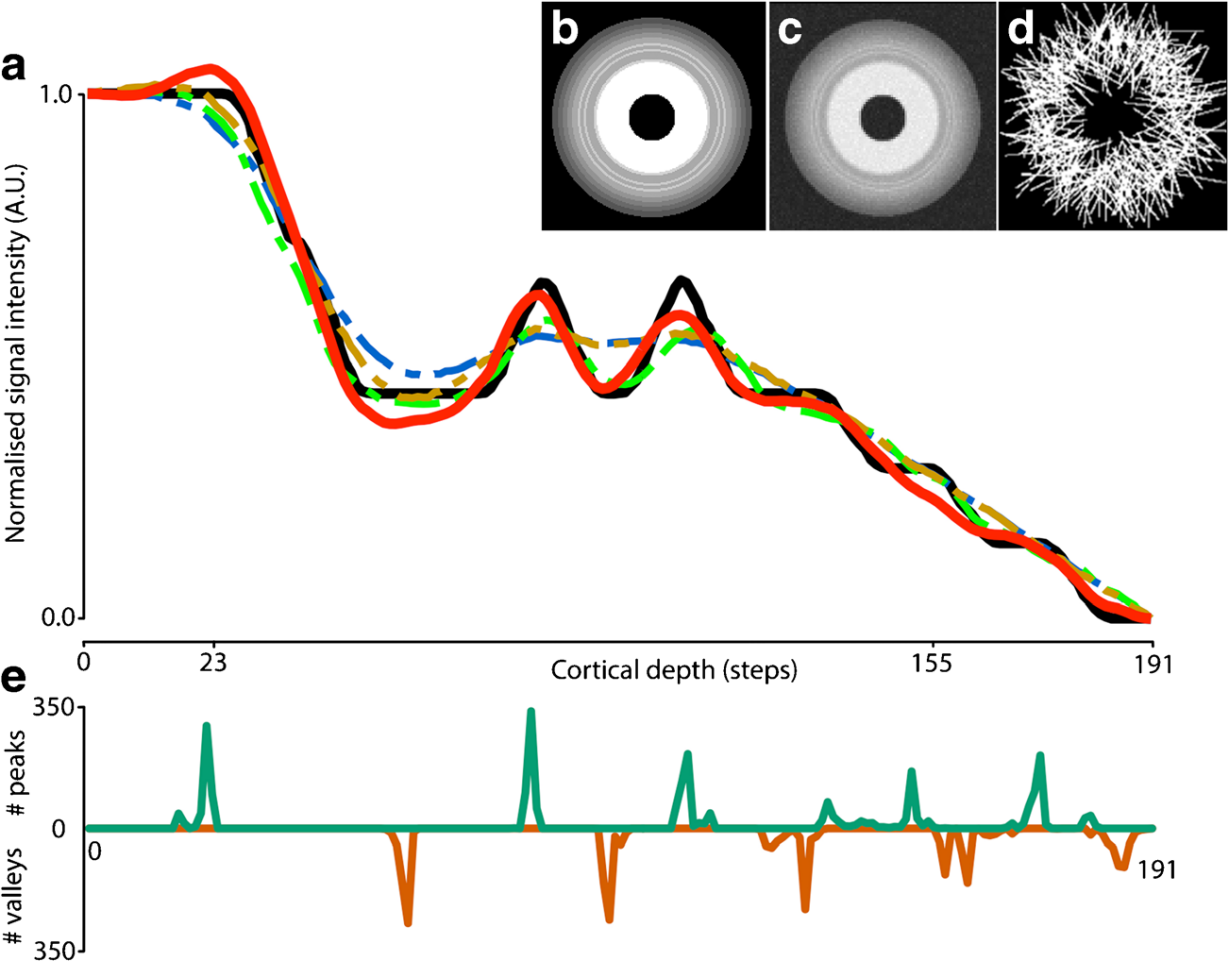
Results of simulation experiment. The black solid line shown in (**a**) represents the ground truth cortical profile and the red solid line represents the computed BAM profile. The intermediate steps are shown with dashed lines: the blue line represents sampling without alignment nor deconvolution, the green line represents alignment only, and the orange line represents deconvolution only. The mid-slice of the noise-free and noisy model volume is shown in (**b**) and (**c**) respectively. The applied realistic sampling scheme is shown in (**d**), in which white lines represent lines along which the profile is sampled, here in 360 samples with normal distributed random variation in coordinates of both begin and endpoints. (**e**) displays distributions of peaks and valleys positions detected from the bootstraps samples for the computed BAM profile (the red solid line)

To assess the feasibility of the method we created a 3D spherical model (isotropic voxel size 0.5 mm, 200x200x200 voxels) of the cortex serving as ground truth (Fig. 2a). A volume was created with a sphere containing 10 consecutive shells with different intensity values (800, 700, 600, 680, 600, 680, 600, 550, 450, 400, respectively) and a background value of 200 to simulate a cortex with two high signal intensity bands. A cross section of this cortex model is shown in Fig. 2b.

The ground truth cortical profile (the dashed black line) was computed by averaging 360 equally distributed (with a 1 degree interval) profiles within the middle slice (slice #100). To simulate scanner-induced noise the model was blurred with an isotropic 3D Gaussian blurring kernel (sigma = 1 mm) followed by down-sampling (1 mm isotropic voxels) after which Rician noise was added (SNR 10% of the background signal). The result is shown in Fig. 2c.

To mimic variations due to suboptimal pial and/or white matter boundary detection, the begin and end coordinates of the 360 individual profiles were varied by adding an offset (modelled with Gaussian noise, sigma 0.2 mm) to the in-plane coordinates of the individual profiles yielding the second sampling scheme shown in Fig. 2d. The 3D deconvolution step used in the computation of the average profiles (for both sampling schemes) was identical to the one described above. Note that, although the profiles were sampled from a single (2D) slice, we must use a 3D model to be able to apply the 3D deconvolution step. To generate the BAM profile (the red solid line) and to assess the stability of the result, bootstrapping was applied as described above.

To assess the contribution of the deconvolution and alignment step separately, BAM profiles were also generated with the application of alignment only or deconvolution only.

### Participants

Three healthy volunteers (all male, mean age 28.8 years (SD = 1.0)) participated in the study after having signed informed consent. All experimental procedures were conducted in accordance with the 1964 Declaration of Helsinki (most recently amended in 2008, Seoul), and approved by the ethics committee of the University Medical Center Utrecht.

### MRI Image Acquisition

The 3 T MRI T1-w data were acquired using a 3 Tesla Philips Achieva scanner (Philips Healthcare, Best, Netherlands), with an 8-channel head coil, and a 3D MPRAGE sequence (number of excitations per inversion 180; TR/TE 10 ms/4.6 ms; flip-angle 8°; FOV 240x240x160 mm; 200 slices, 0.8 mm isotropic voxel size; SENSE parallel imaging factor 1.4 (RL) and 1.7 (AP) in both phase-encoding directions; inversion time 964.4 ms; gradient non-linearity correction; total scan duration 602 s), hereafter referred to as 3T-T1.

The 7 T MRI T1-w data were acquired using a 7 Tesla Philips Achieva scanner (Philips Healthcare, Best, Netherlands) with a 32-channel receive head coil (Nova Medical, Wilmington, MA, USA). First, a conventional T1-w 3D–MPRAGE scan was acquired (voxel size 0.8 mm isotropic, number of excitations per inversion 300; TR/TE 7 ms / 2.9 ms; flip-angle 8°; inversion time 1200 ms; time delay between inversion pulses 3500 ms; FOV 200x250x190.4; acceleration using SENSE 2.8; 238 slices; total scan duration 296.3 s). This scan has the same resolution and a similar contrast to the scan acquired at 3 T and served as an ‘intermediate’ scan to register the 3 T scan with the 7 T scans. Second, a 3D proton density (PD) scan was acquired (voxel size 1.0 mm isotropic, TR/TE 5.7 ms / 2.8 ms; flip angle 1°; FOV 200x250x190; SENSE 1.8 (anterior-posterior) × 1.8 (right-left); 190 slices; total scan duration 49 s) which was used to correct the 7 T T1-w scans for B1 field inhomogeneities (Marques et al. 2010; Van de Moortele et al. 2009). Next, three myelin-sensitive T1-w MPRAGE scans were acquired (voxel size 0.5 mm isotropic, number of excitations per inversion 300; TR/TE 7.5 ms / 3.5 ms; flip-angle 8°; inversion time 1200 ms; time delay between inversion pulses 6000 ms; FOV 200x250x180; SENSE 2.5 (anterior-posterior) × 2.5 (right-left); 360 slices; total scan duration 446.3 s, see (Fracasso et al. 2016)). These three myelin-sensitive scans were aligned and averaged to increase SNR. No correction for gradient non-linearity was applied for the 7 T scans. To increase homogeneity, this average scan was then divided by a smoothed version (12 mm FWHM Gaussian kernel) of the PD scan resulting in one scan to which we hereafter refer to as MS7T.

### 7 T to 3 T Image Registration

Because the 3T-T1 and MS7T scan differ in contrast and may be slightly deformed with respect to each other, as they were acquired on different scanners, the intermediate scan (see above) was used to optimise the registration. The registration was performed using ANTs (Avants et al. 2011) and prior to the registration all scans were corrected for effects of B0 inhomogeneity using N4 (Tustison et al. 2011). To correct for possible head motion between scans, first, a linear (rigid and affine) transformation was computed that registers the intermediate scan to the MS7T scan using mutual information as optimisation metric. Next, the intermediate scan was registered to the 3T-T1 scan using a combination of linear (rigid and affine) registration with cross correlation as optimisation metric followed by nonlinear (SyN) warping with cross correlation as optimisation metric. The transformations were combined and then used to align the MS7T to the 3T-T1 scan, using Hamming windowed sinc interpolation.

### Cortical Segmentation and Surface Construction

FreeSurfer was used for a cortical segmentation of and automatic inner (white/grey matter) boundary and outer (pia mater) boundary delineation in 3 T data (v5.3.0, http://surfer.nmr.mgh.harvard.edu/) (Fischl 2012). The results were visually inspected and manually corrected if needed. In order to use the inner and outer boundaries that were computed from the 3T-T1 scan to sample the MS7T scan the latter was registered towards the former.

Regions of interest were created by default by FreeSurfer’s recon-all pipeline. The thresholded Brodmann Area annotation (except for V7) from FreeSurfer was used (Fischl et al. 2008). The distinction of these areas in the atlas and annotation relies on information from ten post-mortem brains, and made generalizable to other brains on basis of cortical folding. Note, however, that cytoarchitecture cannot be measured with MRI but that classification of cytoarchitecture can only be inferred via cytoarchitecture-myeloarchitecture correspondence, and is especially difficult in long stretches of cortex such as BA 1,2,3 and 4.

### Contribution of Deconvolution and Alignment

The method combines deconvolution and profile realignment. To investigate the effect of the different processing steps, the automatic procedure was applied with and without deconvolution of the volume, and with and without parametric time warping (ptw) alignment of the profiles, to the 3T-T1 and MS7T scan for one subject (PP3) in the left V1 region.

### Clustering

To quantitatively assess the reproducibility for BAM profiles computed from the 3 T with respect to the 7 T data, we perform two different analyses. In the first (fully data-driven) analysis we use a hierarchical crisp clustering algorithm based on the dynamic time warping distance (DTW) implemented in dtwclust (https://cran.r-project.org/ package = dtwclust). The aim is to determine to what extent the 7 T BAM profiles contain information specific to the various brain regions and to determine if clustering of the 3 T data produces similar results. The DTW distance is a stretch-insensitive measure of the ‘inherent difference’ between two profiles (Giorgino 2009), operating on information from the complete BAM profiles. An advantage of using DTW-based clustering is therefore that it does not require us to define salient features beforehand, making these results more generalizable. This type of clustering does however require us to determine the number of clusters beforehand. Because the primary goal is to determine to what extent the BAM profiles computed from 3 T scans contain similar information to the 7 T BAM profiles we decided to use the GAP statistic to compute the optimal number clusters for the BAM profiles computed at 7 T. The cluster pattern obtained for the 7 T BAM profiles will then serve as ground truth for the clustering results obtained with the same number of clusters for the 3 T data. The amount of overlap between 7 T and 3 T cluster patterns is then computed using the adjusted Rand index (ARI) (Rand 1971; Vinh et al. 2010). The ARI is bounded above by 1, and scaled in such a way that 0 represents the amount of overlap obtained by chance. Please note that the ARI is not bounded at 0 but can be negative as well.

To determine the level of significance of the amount of overlap we use randomization sampling with replacement (1000 samples for the 3 T clustering) to compute a distribution for the bootstrap ARI values under the null hypothesis. If the ARI of the original cluster pattern is larger than 95% of the ARI values in the distribution then the overlap is deemed significant). We consider this an indication that 3 T BAM profiles are indeed similar to the BAM profiles computed at 7 T. Note that here we use randomization testing with replacement instead of permutation testing. For each sample, the original BAM labels are assigned to 42 profiles that are randomly selected (with replacement) from the original set of 3 T BAM profiles after which clustering is applied and the ARI between the resulting clustering pattern and the 7 T clustering pattern is computed. The reason for this is that the computed distance function for clustering is invariant under permutation of the profiles and as a consequence the same profiles will form the same clusters, although the cluster number itself may change. Of further note is that because ARI is not a true distance measure (Vinh et al. 2010) we cannot use it to compute for instance effect sizes or to directly compare clustering results from 7 T data and 3 T results with respect to a third ground truth clustering.

### V1 Versus V2 Versus V7 Comparison

In the second analysis we do use a priori knowledge on differences between V1 and V2 as well as known differences in myelin concentration between higher order (V7) and lower order (V1, V2) visual cortices. The major difference between adjacent areas V1 and V2 is that layer 4 in V1 is heavily myelinated (the line of Gennari) (Glasser and Van Essen 2011). This difference is often used to assess myelin-sensitive MRI scan sequences (Fracasso et al. 2016). Here we investigate if this difference (expected to be maximal at approximately the center of the profiles) can be detected both in 7 T and 3 T data. For this we compute the pairwise distance (V1 - V2) at the center of the profiles (X = 50) and compute if it is significantly larger than zero for both 7 T and 3 T data.

In contrast to primary visual regions, higher order visual regions like V7 have a lower myelin content (Glasser and Van Essen 2011). For cortical profiles computed from myelin-sensitive MRI scans this should result in relatively fast drop-off in signal intensity in the direction from the white matter boundary towards the pial boundary. Here we will measure the differences between the V7 profile and the primary visual regions V1 and V2 at the same position (X = 50) as in the previous comparison. Before assessing the intensity value of a BAM profile at that position, the BAM profiles were first aligned to each other.

### Gibbs Ringing

One concern may be that the peaks or valleys in the average profiles are not linked to myeloarchitectural properties but that they are in fact the product of Gibbs ringing as these artefacts show up as decaying bands in the vicinity of sharp transitions in the image (e.g. cortex boundaries) and could be misinterpreted as laminar information. Gibbs ringing is not only of concern in post-processing (in the deconvolution step) but especially in image acquisition.

For acquisition-related Gibbs ringing the ‘size’ of the rings (that is, the frequency of the decaying over- and undershoot) is directly related to the voxel size of the original scan (Haacke and Brown 1999). Because the voxel size of the MS7T scan is 0.5 mm isotropic and 0.8 mm isotropic for the 3T-T1 scan one would expect that artificial peaks (and valleys) due to Gibbs ringing would be systematically found at different locations along the cortical depth when comparing the average profiles between the 3 T and 7 T scans. To determine if there is a systematic difference in peak locations between 3 T and 7 T average profiles we computed the distance for two different peaks with respect to the rightmost valley (distance I and distance II in Fig. 6) in V1. Values for distance I and distance II are computed for both 3 T and 7 T profiles. If these values show a significant difference between 3 T and 7 T (computed using a paired t-test) then this is an indication that peaks (or valleys) are due to acquisition-related Gibbs ringing.

## Results and Discussion

### Simulation Experiment

In Fig. 2 the results of the simulation are shown to demonstrate the feasibility of the method. Simply averaging without realignment or deconvolution (the blue dashed line) results in a low-detailed BAM profile in which the two separate high-intensity bands seen in the model (black solid line) cannot be identified. Averaging without realignment (but with deconvolution) is depicted by the orange dashed line while the result of averaging with realignment but without the deconvolution step is depicted by the green dashed line. The red solid line denotes the BAM profile computed using the proposed method. In this average profile two peaks can be detected which coincide with the high intensity bands in the model. Note, however, that in the BAM profile an additional small peak is found on the left.

The distribution of the peaks and valleys computed for each of the 500 bootstrap samples that were used to generate the BAM profile are presented in Fig. 2e. These peak and valley distributions give us information on which of the peaks and valleys in the BAM profile can be reliably detected and may be linked to true physical properties of the cortex. Note for instance that for this simulation there is virtually no overlap between peaks and valleys suggesting that their positions are relatively constant and they could therefore serve as salient features.

Comparing the average model profile (black solid line) with the reconstructed BAM profile (solid red line) suggests that no artificial peaks or valleys are introduced. However, we do see a slight over- and undershoot of the signal in the immediate vicinity of a sharp signal change (at depth X = 23). This shows that one should interpret peaks and valleys adjacent to a very large signal intensity change (for T1-w contrast typically the pial or white matter boundary) with caution. Besides this peak, no other spurious peaks or valleys were found, indicating that the effects of Gibbs ringing due to deconvolution are limited.

From the ground truth (the black solid line) it can be seen that there are three small peaks and two small valleys in the declining slope on the right side of the profile, with X = 155 at its centre. The reconstructed BAM profile actually fails to show the middle of three small peaks (i.e. a false negative) although it can be observed from the peak/valley distribution (panel e) that the peak is found in a considerable number of the bootstrap profiles (the second green peak from the right). Apparently, this small peak is averaged out in the BAM profile suggesting that, although the method can be used to detect the larger maxima, more subtle details may still be missed.

We note that (especially for the in vivo data) a one-to-one correspondence between peaks and valleys over the bootstrap profiles may not exist as the number of peaks and valleys per bootstrap profile may vary. Therefore, it is not straightforward to use the peak/valley distributions to calculate for instance confidence intervals for a specific peak or valley location in the BAM profile and for this reason we just present the histograms of peaks and valleys found.

### Contribution of Deconvolution and Alignment

**Fig. 3:**
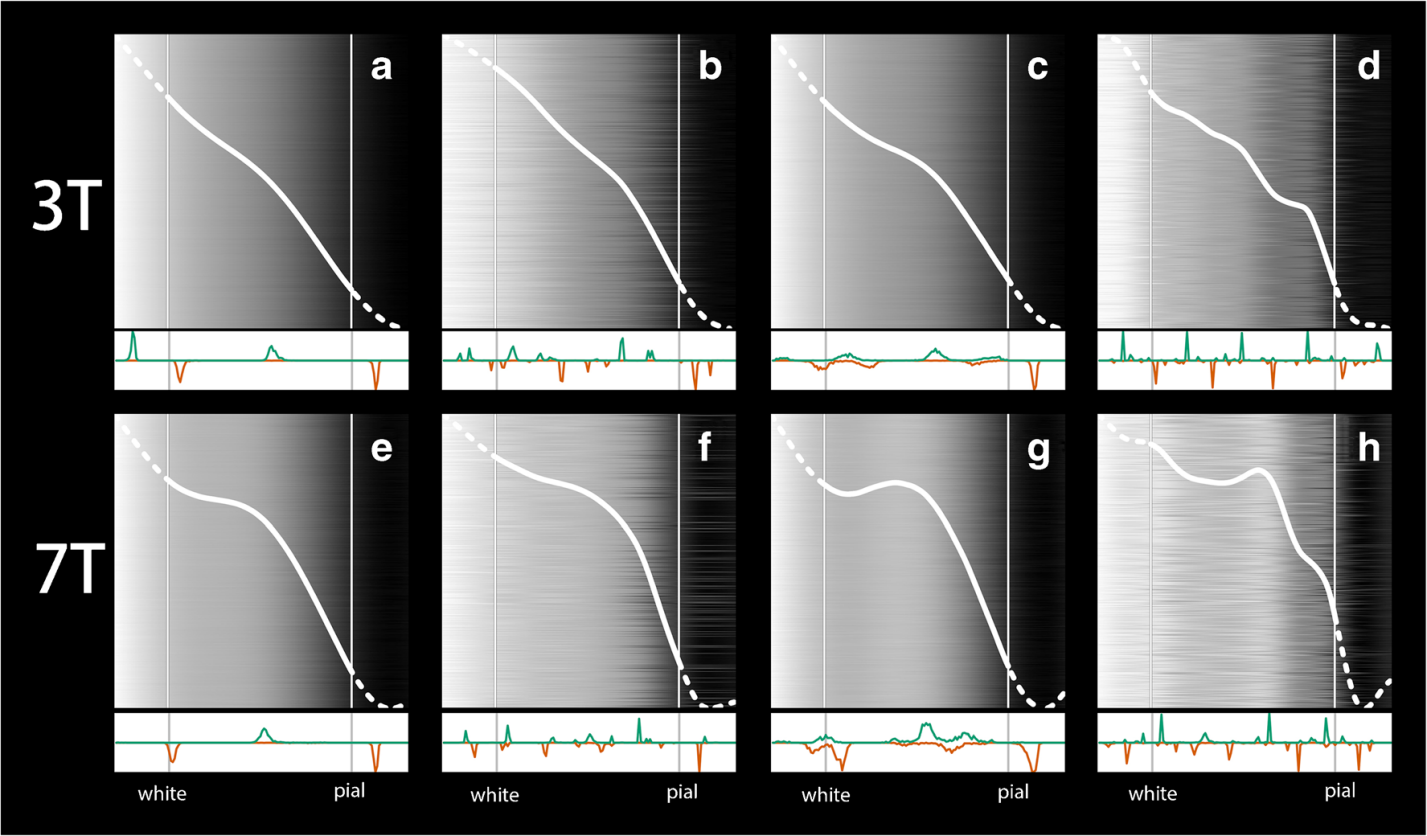
Results of method applied on in vivo data in V1. Top row displays 3 T results, with displaying BAM profiles and corresponding peak and valley histograms of (**a**) non-deconvoluted nonaligned data, (**b**) aligned, non-deconvoluted data, (**c**) non-aligned, deconvoluted data; (**d**) aligned deconvoluted data; bottom row (**e**)–(**h**) depict result of identical processing, but for 7 T data. Note that the histograms are scaled to the maximum number of peaks or valleys

The results of applying the automatic procedure to the left V1 for subject 3 (PP3) with and without deconvolution, and with and without alignment, and applying the bootstrap analysis are shown in Fig. 3. The BAM profiles computed with ptw alignment only (Fig. 3b and f) show a more pronounced local maximum for both the 3T-T1 and MS7T scans (with MS7T showing a more pronounced local maximum than 3T-T1) than the BAM profiles computed without deconvolution or ptw alignment (Fig. 3a and e). Figure 3c and g show the results when averaging is done over the profiles extracted from the deconvoluted scans but without the ptw alignment step. Especially for MS7T this appears to result in a more pronounced curvature of the BAM profile in comparison to the BAM profiles shown in Fig. 3a and b. Finally, Fig. 3d and h show the BAM profiles computed with deconvolution and ptw alignment. Both figures show more detail (e.g. multiple peaks) suggesting that it is indeed the combination of deconvolution and realignment that leads to substantial more detail in the computed BAM profiles. However, similar to the profiles shown in 3a–c and 3e–g it is evident that the peaks are more pronounced in Fig. 3h in comparison to 3d, suggesting that the 7 T data is (as expected) of better quality. Note that the peaks may be displaced when comparing the rightmost column (where both alignment and deconvolution are applied) with the other profiles in the other columns. This is because the best reference profiles (chosen for the alignment in each bootstrap) are likely to differ (both in location and scaling) between the BAM profiles shown in the rightmost column (where alignment is applied) and the rest. The location of a best reference profile with respect to the cortex is determined by the estimated inner and outer cortical boundaries computed with Freesurfer, which may not always be completely correct. Although the effect of the chosen reference profile on the shape of final BAM profile is limited (thus limited model shine-through) this is most likely not the case for the absolute location and size of the BAM profile. This also applies to the comparison of BAM profiles from 3 T versus 7 T.

When comparing results shown in Fig. 3 with the simulation (Fig. 2) one can see that in the simulation the various computed profiles do line up with the ground truth. However, the presented simulation is a rather basic simulation and is included to show the feasibility of the proposed method and to show that effects of Gibbs ringing on the final BAM profile are limited. The fact that the peaks found in the simulation using the full procedure are nicely aligned with the ground truth may actually reflect the basic nature of the simulation. The noise in the in vivo data may be of a more complex nature and the variation in laminal makeup within a Brodmann area is likely larger than zero (as is assumed in the simulation). For example, in the simulation experiment alignment without deconvolution results in a fairly good (albeit less pronounced) approximation of the ground truth while in the in vivo data (shown in Fig. 3) this is clearly not the case. We further note that we chose not to realign the bootstrap results before averaging them to compute the final BAM profile although such a ‘second-level’ realignment step could be included. The motivation for this choice is that the histograms (shown at the bottom of each panel) now also include the inherent variability of location of the model profiles.

### Average Profiles 7 T and 3 T Data

**Fig. 4:**
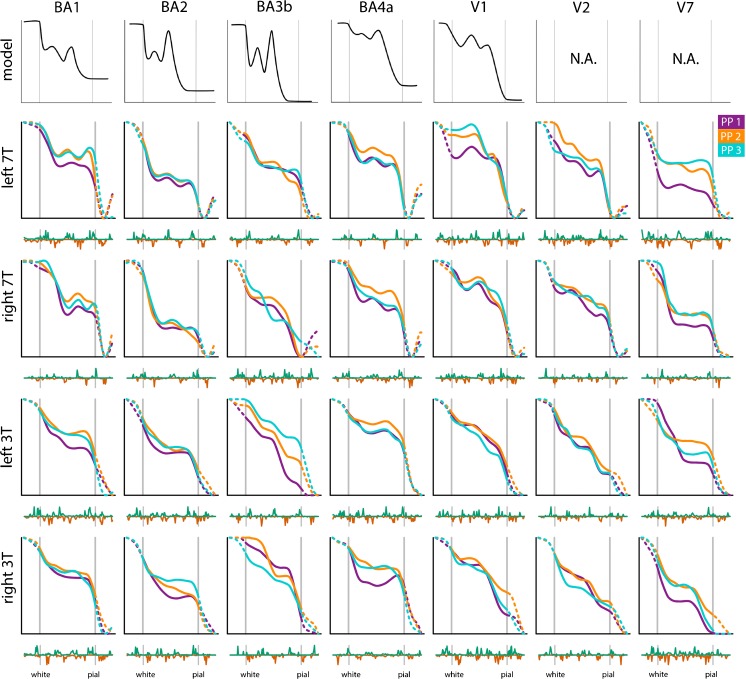
Results from seven cortical areas. BAM profiles of areas BA1, BA2, BA3b, BA4a, V1, V2 and V7. The model profiles for BA 1, 2, 3b and 4a are provided as an indication of the expected myelin concentrations and were based on the model profiles presented in (Dinse et al. 2015), where PP denotes participant. Here both x- and y-axis of these model profiles are inverted to match the signal intensity and cortical depth measurement in our data. Original figures are provided as supplementary Fig. S1 (V1) and S2 (BA1–4). Note that the left V1 profile shown for subject #3 is a scaled version of the profile as shown in Fig. 3h in order to align it with the corresponding profiles for subjects #1 and #2

In Fig. 4 the BAM profiles computed for the cortical areas BA1, BA2, BA3b, BA4a, V1, V2 and V7 are shown for all subjects and for both MRI field strengths. For four regions (BA1, BA2, BA3b, BA4a), a theoretical T1 model profile signal is available (Supplementary Fig. S2; (Dinse et al. 2015)) shown in the top row of the image for reference purposes. Dinse et al. (2015) used prior knowledge on both cytoarchitecture (e.g. cell density) and myeloarchitecture (local myelin content) to model a T1-signal profile per brain area. For V1 a reference profile is provided that is based on high resolution post-mortem MRI (See Supplemental Fig. 1). For V2 and V7 no reference profile is available. Note that because of the use of a best representative profile as target profile for alignment the absolute positions of peaks and valleys found in BAM profiles computed for the same area may differ between subjects and scanners. The comparison of BAM profiles should therefore be made on relative features (e.g. relative distances between minima and/or maxima).

Most of the BAM profiles display multiple peaks and valleys. The general impression is that, except for BA1 and BA2, the middle peak is present in the BAM profiles both at 7 T and at 3 T. Particularly in the average profiles computed for V1 acquired at 7 T, where this middle peak appears to be more pronounced compared to other cortical areas. This is in concordance with the model profile obtained from the work from Dinse and colleagues.

We do, however, recognize that besides similarities there are also marked differences between the profiles computed for 3 T and 7 T data. For example, for BA1 in the left hemisphere we see two peaks in the 7 T profiles in subjects #2 and #3 while there is only one peak in the corresponding 3 T profiles. For the right hemisphere in BA3 the profiles for 3 T and 7 T data look different, especially for subjects #1 and #2. For BA2 (left hemisphere) the 7 T profiles show more detail than the 3 T profiles. A similar observation can be made for BA4a, were especially for subject #3 the middle peak cannot be detected for 3 T while for 7 T the middle peak can be clearly detected for all three subjects. Overall, it appears the profiles computed 3 T and 7 T data are more similar for the left hemisphere than for the right hemisphere. The histograms computed for 7 T suggest that, for most areas, peaks and valleys occur in groups of four. For the 3 T histograms the pattern is less consistent. In sum, this qualitative comparison clearly suggests (as could be expected) that the profiles computed for 7 T are more detailed and more consistent over subjects and hemispheres than the profiles computed for 3 T data.

Between 20 and 25% of the FreeSurfer profiles are selected for processing due to the thickness and curvature constraints. For detailed information on the initial number of FreeSurfer profiles and the selected number of profiles per region, see supplementary Table S1.

### Clustering Results

**Fig. 5:**
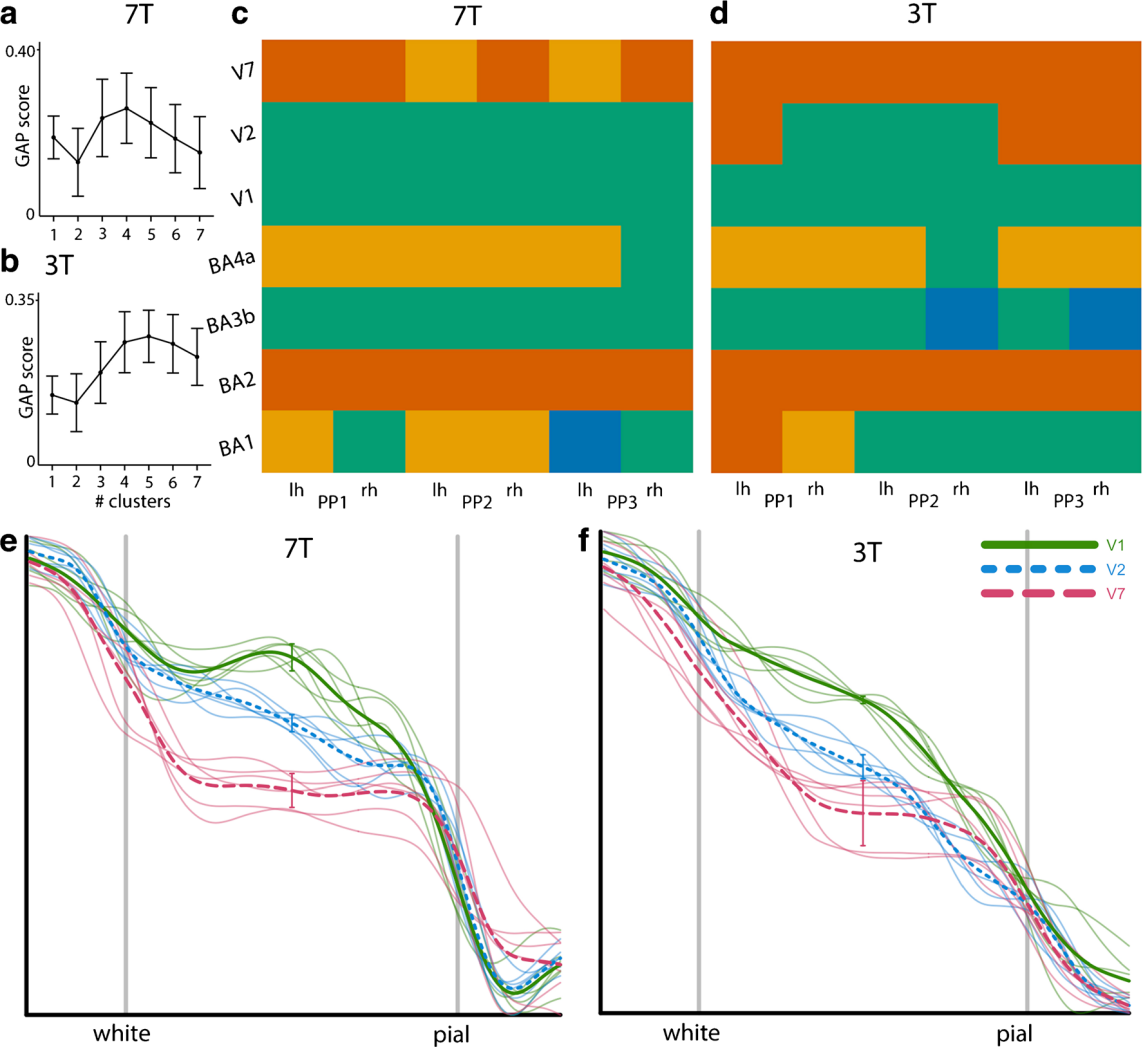
Results from crisp clustering with dynamic time warping distance. In (**a**) the GAP score plots are shown for both 7 T and 3 T data. Clustering results (with k = 4) for BAM profiles computed from seven areas, for 7 T data and 3 T data are shown in (**b**). The colour of each block indicates the belongingness of each BAM profile to which cluster. BAM profiles are presented according to participant (PP) and left and right hemisphere (lh, rh) per column, and according to area per row. (**e**) displays BAM profiles from three participants, two hemispheres, of V1, V2 and V7, at 7 T. Error bars indicate +/− 1 SD at the centre of the profile. (**f**) displays similar BAM profiles, but at 3 T

Maximum global GAP statistics computed for 7 T data suggest that the best number of clusters is 4 (See Fig. 5a). The results for the hierarchical dtw-based clustering of the 7 T and the 3 T data, using four clusters (k = 4), are shown in Fig. 5b. The ARI computed for 3 T clustering with 7 T clustering as the reference is 0.270, which is significantly larger (*p* < 0.0005) than zero (reflecting the amount of overlap by chance) suggesting that indeed 3 T and 7 T profiles bear similar information. We note for the sake of completeness that the ARI computed between the 7 T and 3 T clustering patterns with seven clusters (k = 7) is 0.147 (1000 randomization samples; *p* = 0.011), which is still significant. When assessed with permutation testing, the significance level for ARI computed with four clusters reaches *p* < 0.00005 and with seven clusters *p* = 0.003.

From the clustering pattern for 7 T presented in Fig. 5c it can be seen that areas V1, V2 are grouped in the same cluster implying that these areas have similar features and contain similar information. Indeed, from previous literature it is known that the areas are similar in terms of local myelin content and that the main difference between V1 and V2 is the presence of the line of Gennari in V1. The question is whether this difference can be reliably detected and used to further distinguish between V1 and V2 at 7 T, and to what extent this can be done at 3 T. Note that BA3b is also part of this cluster, and is like V1 and V2 also a primary sensory cortex. We further observe that V7 is clustered together with BA2. From the reference profiles shown in Fig. 4 it is not immediately clear which of the primary somatosensory regions (BA1, BA2, BA3b) has a higher myelin concentration but from the profiles plotted in Fig. 4 it appears that indeed BA2 has a lower overall T1 signal compared to BA1 and BA3b. Primary motor cortex BA4a appears to form a separate cluster. This is in concordance with information from the reference profiles where it can be seen that BA4a is expected to have the overall highest T1-signal over the profile. These observations hold to a large extent for the clusters computed for the 3 T scans. However, one should keep in mind that the fact that the cluster pattern for 3 T are similar to the 7 T cluster pattern does not imply that the clusterings are based on the same profile features. Dynamic time warp clustering is not based on salient features (e.g. peak and valley positions) but takes the complete profile into account. Therefore, we cannot rule out that different weightings of profile features for 3 T and 7 T could still lead to a similar clustering.

### V1 Versus V2 Versus V7 Comparison

The signal difference between V1 and V2 measured at the middle (X = 50; Fig. 5e/f) of the BAM profiles per subject and per hemisphere is significant for both 7 T data (t(df = 8.4) = 10.1; *p* < 0.00001) and 3 T data (t(df = 5.9) = 13.1; *p* < 0.00002). The signal difference between V7 and V2 measured at the same position is also significant for both 7 T data (t(df = 7.4) = 8.8; *p* < 0.00005) and 3 T data (t(df = 6.3) = 3.3; *p* < 0.015). In sum, the results from both analyses strongly suggest that indeed profiles computed from 3 T scans bear information similar to 7 T profiles albeit that the differences between profiles for the V1, V2 and V7 are more pronounced in the 7 T data.

### Gibbs Ringing

Acquisition-related Gibbs ringing in the MS7T volume has been previously assessed by Fracasso and colleagues (Fracasso et al. 2016) by drawing profiles through white matter at a white-gray matter boundary. The authors based their assessment on the rationale that if profiles sampled through gray matter show maxima that were due to Gibbs ringing, that same ringing would have to be present in cortical profiles sampled from white matter. Based on their results Fracasso and colleagues concluded that Gibbs ringing could not explain the reported peaks in gray matter.

**Fig. 6:**
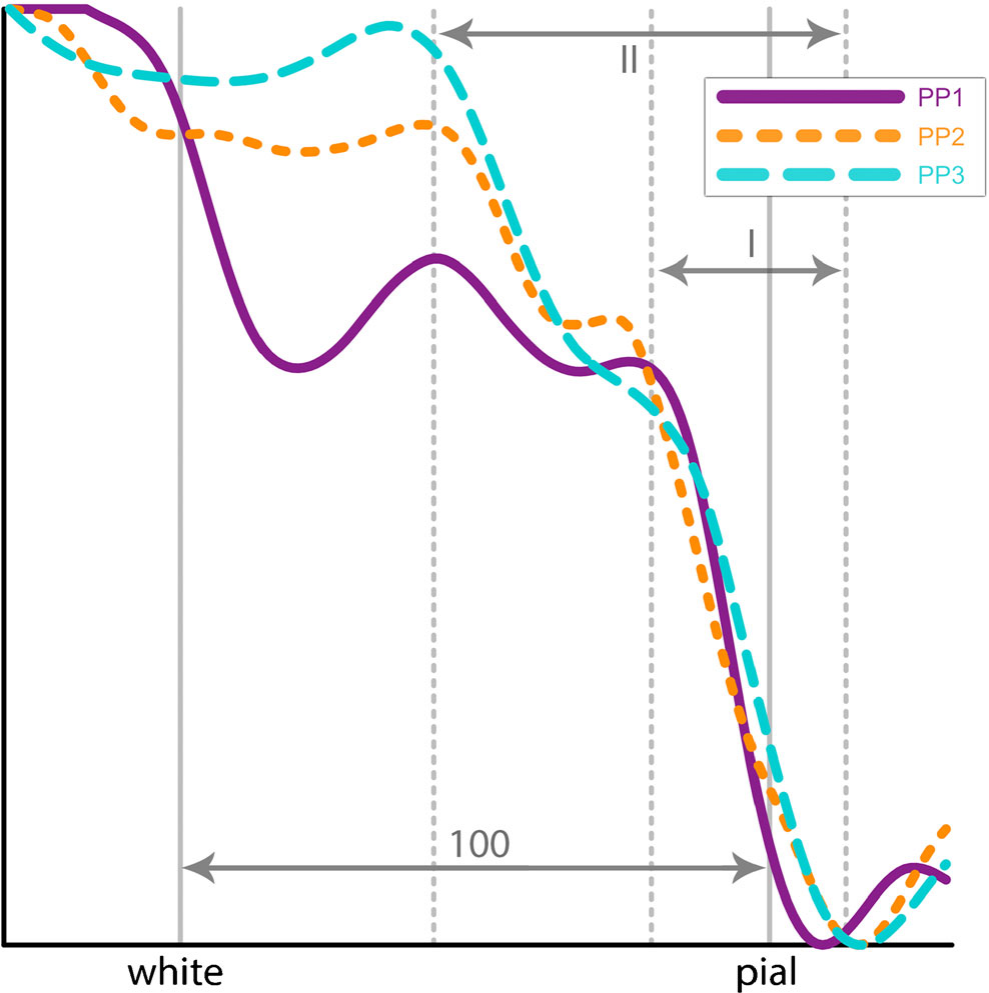
Overview of distances used in assessment Gibbs ringing. This figure depicts the distances I and II, as measured in x-steps, divided by the total 100 steps between white-gray matter and pial boundary, between the rightmost minima and first two peaks to the left of that minima. Results are shown in Table 1

Here we performed an additional analysis to investigate the possible role of acquisition-related Gibbs ringing. Based on the bootstrap results for peaks and valleys locations two distances (denoted I and II in Fig. 6) are computed between two peaks and the rightmost valley for the average profiles of V1. The results are reported in Table 1. To take possible scaling differences into account we also compared the fractions I/II computed for 3 T and 7 T scans (combining left and right regions). The mean distances for I and II (left and right combined) are for 3 T: 0.19 (SD = 0.03), 0.62 (0.10) and for 7 T: 0.18 (0.04) and 0.54 (0.09) respectively. The mean fraction I/II computed for 3 T scans is 0.31 (SD = 0.03) and for 7 T 0.33 (0.44). The results for the paired t-tests between 7 T and 3 T data are *t* = 0.39, *p* = 0.71 for distance I, *t* = 0.96, *p* = 0.39 for distance II and *t* = −0.20, *p* = 0.85 for fraction I/II. Note that that these measures are all relative measures (distances or fractions between distances) and are therefore invariant to possible effects of model shine-through.

**Table 1:** Distance measures

Subject	Volume	Hemisphere	Distance I	Distance II	Ratio II/I
1	3 T	Left	0.21	0.57	0.3684211
		Right	0.18	0.63	0.2857143
	7 T	Left	0.14	0.53	0.2641509
		Right	0.21	0.61	0.3442623
2	3 T	Left	0.25	0.62	0.4032258
		Right	0.22	0.61	0.3606557
	7 T	Left	0.14	0.36	0.3888889
		Right	0.19	0.57	0.3333333
3	3 T	Left	0.21	0.59	0.3559322
		Right	0.16	0.54	0.2962963
	7 T	Left	0.18	0.51	0.3529412
		Right	0.29	0.68	0.4264706

The absence of a significant difference itself can of course not serve as proof for a limited effect of acquisition-related Gibbs ringing. In particular because it can be argued that the statistical power (*n* = 6) is very low. But as the difference in distance in case of Gibbs ringing should be around 38% (voxel size 0.5 vs 0.8) one could expect that it can be detected with these small group sizes. Therefore, we think that in combination with the evidence put forward in (Fracasso et al. 2016) the fact that there is no significant difference in the various distances computed from 3 T and 7 T volumes with different voxel resolutions may at least serve as an indication that these peaks are not the product of acquisition-related Gibbs ringing.

### Limitations

The central question in this study was: “Are cortical profiles computed from conventional 3 T scans using the proposed method (to a certain extent) similar to cortical profiles computed from this particular 7 T MRI acquisition that has previously been validated with histology?” and the generalizability of the results presented herein is therefore limited to these particular scans. However, we do not want to imply that this particular 7 T scan acquisition is the best possible solution available to study cortical myeloarchitecture (and is the ground truth) as the quality of a scan does not only depend on the main magnetic field strength of the scanner but is the result of a complex interplay of many factors, including the use of high-performance RF coils, characteristics of the gradient system and chosen image contrast. The fact that a 3 T profile is similar to 7 T profile does therefore not automatically imply that the 3 T profile is free of artefacts, for instance, as both scans are based on T1-weighted contrast and therefore T1-related artefacts common to both scan types may remain unnoticed.

Although the results of the quantitative analyses presented here suggest that the proposed automatic method indeed yields similar cortical profiles for 3 T compared to 7 T, we do recognise that at the individual profile level marked differences between 3 T and 7 T are found. The general impression from the qualitative analyses is that the 7 T profiles are more pronounced (showing clearer peaks and valleys) and are more consistent over subjects and hemispheres. This could be expected as the 7 T scans are not only of higher resolution but are also optimised for contrast within the cortical gray matter. Thus, if possible, the obvious choice is to use a dedicated MRI acquisition to investigate cortical myeloarchitecture. However, if this is not possible (because of the large number of subjects involved or because the scans already have been acquired) then the proposed method may be of use to study (to a certain extent) cortical myeloarchitecture at a group level. Furthermore, we would like to stress that we do not claim that the proposed method measures cytoarchitecture and that the proposed method will have the same limitations that apply for T1-weighted MRI when used to study myeloarchitecture.

### Methodological Considerations

Although the results of these initial experiments are promising several aspects of the method require further investigation. For instance, in the current implementation an isotropic Gaussian kernel with a fixed size (in voxels) was used in the deconvolution step. It can be argued that this point spread function itself does not adequately describe the effects of scanning at a lower image resolution and that other types of deconvolution kernels (e.g. different shapes, sizes) may be more appropriate. We note, however, that here our goal was not to model the effect of differences in scanner resolution per se but merely to increase image detail. The use of a Gaussian blurring kernel has the advantage that it mitigates the potential problem of introducing Gibbs ringing artefacts when applying deconvolution (Chandrawansa et al. 2000). Initial experiments (data not shown) suggested that the chosen kernel parameters are adequate for this proof of concept.

For the other parameters for the Wiener filter Landweber parallel deconvolution algorithm (besides the choice of the PSF) we chose the provided default settings but other settings may improve the results further. For example, here we chose to disable the preconditioner which may lead to a speed up of convergence but could introduce artefacts. The application of the anti-ringing step is merely done as a precaution to limit spurious effects caused by features in the close vicinity of the volume’s boundaries.

The resolution of the 3 T T-weighted scans in the current study is relatively high (0.8 mm isotropic) in comparison with scans typically acquired in large scale studies (e.g. 1 mm isotropic) and one could question if the proposed method would work for lower resolution scans. But successful application of the method will probably not only depend on resolution but for instance also on SNR and T1-weighting. We did however perform initial experiments (Supplementary Fig. S3), suggesting that resolution itself appears not to be a critical factor and the method can be applied in scans with lower resolutions.

The proposed method averages over sets of cortical profiles but here we did not address the issue of optimal set size. Determination of the optimal set size is not straightforward as it depends amongst others on the homogeneity and thickness of the cortical layers in the area of interest, the quality of the cortex delineation, SNR and scanner resolution. If a set is too small then the effects of noise will be insufficiently mitigated. On the other hand, there is the risk that a set is too large. In that case the cortical architecture of the sampled region is insufficiently homogeneous leading to less detail. Further research is needed to determine optimal (and minimal) set size for the various brain regions. Of further note, the sampling of the cortex, which in the current study was done along straight lines, could be improved in future implementations by using sampling schemes that correct for cortical mantle curvature. For instance, corrections based on the heat conduction equation (Annese et al. 2004; Annese et al. 2005). This would increase the number of profiles included per region and as a consequence profiles could be computed for smaller regions enabling the possibility to incorporate more detailed atlases (Glasser et al. 2016; Hagmann et al. 2008).

Deconvolution is usually implemented using a Fourier transformation -- a mathematical operation carried out in the complex domain. MRI data is complex-valued but usually only the magnitude image is stored and the phase image is ignored. This phase image however may contain additional information leading to better deconvolution results. For future studies one may therefore consider to save the phase images as well.

Possible applications of the proposed method can be found in studying the frequently reported cortical thinning in psychiatric diseases. One strategy would be to determine a number of characteristic points (e.g. peaks and valleys; one close to white matter, one approximately at the center of the profile and one close to the pial surface) for the BAM profiles and then define a relative measure based on these characteristic points. For example, the fraction between center to white matter distance and center to pial distance. Then, for ROIs for which group-related differences in thickness were found, a group comparison using this relative measure can be conducted to determine if the effects of thinning are evenly distributed over all layers or whether the thinning can be attributed to the deeper or more superficial layers. We previously outlined such an approach to study effects of schizophrenia (Mandl et al. 2015).

We note that the proposed method may not only be useful to study psychiatric diseases but can also be used to study the developing brain. For instance, brain development is accompanied by widespread cortical thinning (Van Soelen et al. 2012) and the question is if this thinning reflects neuronal pruning (selective removal of neurons and connections) or that it actually reflects white matter encroachment (where the myelin concentration of the deeper layers increases). In case of pruning the cortex actually thins but in case of encroachment the cortex only appears to become thinner as the deep layers show a T1 signal similar to white matter. Applying the proposed method in a longitudinal study design could be used to determine which of the two explanations is correct.

In the current study we focused on the similarities and differences in average cortical profiles computed from T1-w scans but the proposed automatic method is not limited to T1-w contrast per se. Similar to the use of the cortical boundaries computed on the 3T-T1 to process the MS7T scan, the cortical boundaries can be used to process other types of contrast (e.g. multi T1 (T1 with multiple inversion times), T2, T2*, diffusion-weighted) scans from the same subject, provided that an accurate alignment can be obtained. In particular composition analysis based on multi T1 mapping is potentially interesting as it is an alternative way to obtain layer specific information at low resolution and may be combined with the proposed method in the current study (Lifshits et al. 2017).

### Summary

Here we introduced a new automatic method to compute detailed average profiles for cerebral cortical regions from T1-w scans routinely acquired at a clinical 3 T MRI scanner in vivo (3T-T1). To validate this new method, we compared BAM profiles for various cortical regions (BA1, BA2, BA3b, BA4a, V1 and V2) computed from 3 T T1-w scans, with BAM profiles computed from 7 T myelin-sensitive T1-w scans (MS7T). The computed BAM profiles for the 3T-T1 and MS7T scans result in similar clusterings. As expected, a significant higher relative signal at the center of the profile (the approximate location of the line of Gennari) is found for V1 compared to V2 for both 3 T and 7 T data. Moreover, comparison of the BAM profile for V7 (which has a lower myelin concentration than V1 and V2) with the V1 and V2 profiles indeed shows a relatively fast signal drop from the white matter boundary and a significant lower signal at the middle of the BAM profile for both 3 T and 7 T data. Comparison of peak positions in BAM profiles of V1 computed from 3 T and 7 T scans suggests that the peaks have a location that is irrespective of volume resolution and are therefore not the result of Gibbs ringing.

In conclusion, although the exact relation between the information extracted from the average cortical profile and the myeloarchitecture of the cortex requires further clarification, the results of our study suggest that this information obtained at 3 T is in good agreement with information obtained at ultra-high field MRI and can be used to study various architectural aspects of the cortex. The proposed fully automatic method therefore is a step forward to study the myeloarchitecture of the human cortex in vivo using scans routinely acquired on clinical MRI scanners, both in health and disease.

## Information Sharing Statement

The MRI data used in this paper was acquired in the UMC Utrecht and is not part of an online repository. The following openly available software was used: R (RRID:SCR_001905, https://www.r-project.org), AFNI (RRID:SCR_005927, https://afni.nimh.nih.gov/download), ANTs (RRID:SCR_004757, http://stnava.github.io/ANTs/), FreeSurfer (RRID:SCR_001847, https://surfer.nmr.mgh.harvard.edu/fswiki/DownloadAndInstall), FIJI (RRID:SCR_002285, https://imagej.net/Fiji/Downloads), minc-library (RRID:SCR_002391, http://www.bic.mni.mcgill.ca). The in-house developed pipeline is available on request.

## Electronic supplementary material

ESM 1 (DOCX 2.67 MB)
