# Supplementary material for: Detailed T1-Weighted Profiles from the Human Cortex Measured in Vivo at 3 Tesla MRI
Source: Neuroinformatics. 2018 Jan 19;16(2):181–96. doi: 10.1007/s12021-018-9356-2 (PMC5984962; doi:10.1007/s12021-018-9356-2)
Supplement: Supplementary file 1 — (DOCX 2.67 MB) [file 12021_2018_9356_MOESM1_ESM.docx]

**Supplementary Material (3 Figures, 1 table)**

Figure S1


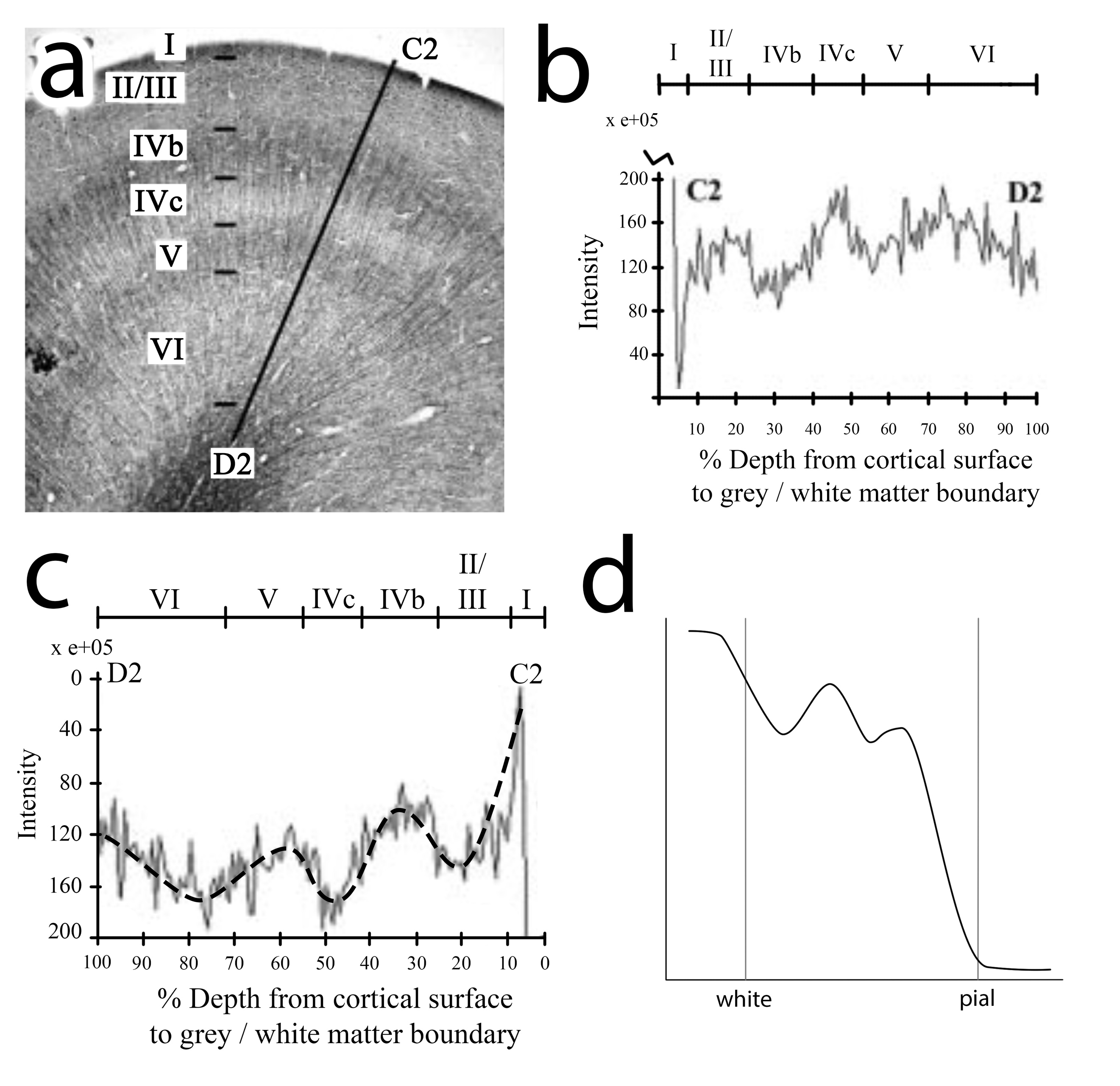


a) shows picture Fig 2 III in Walters et al. (2003), b) shows an excerpt from Fig 2 V in Walters et al. (2003); marked location D2 indicates white matter, C2 indicates pial surface. c) contains the graph from b) mirrored in x and y direction, and overlayed with simplified model line (black dashed). d) contains the simplified model line from c), with extrapolation, to be able to be used in a comparison.

Figure S2


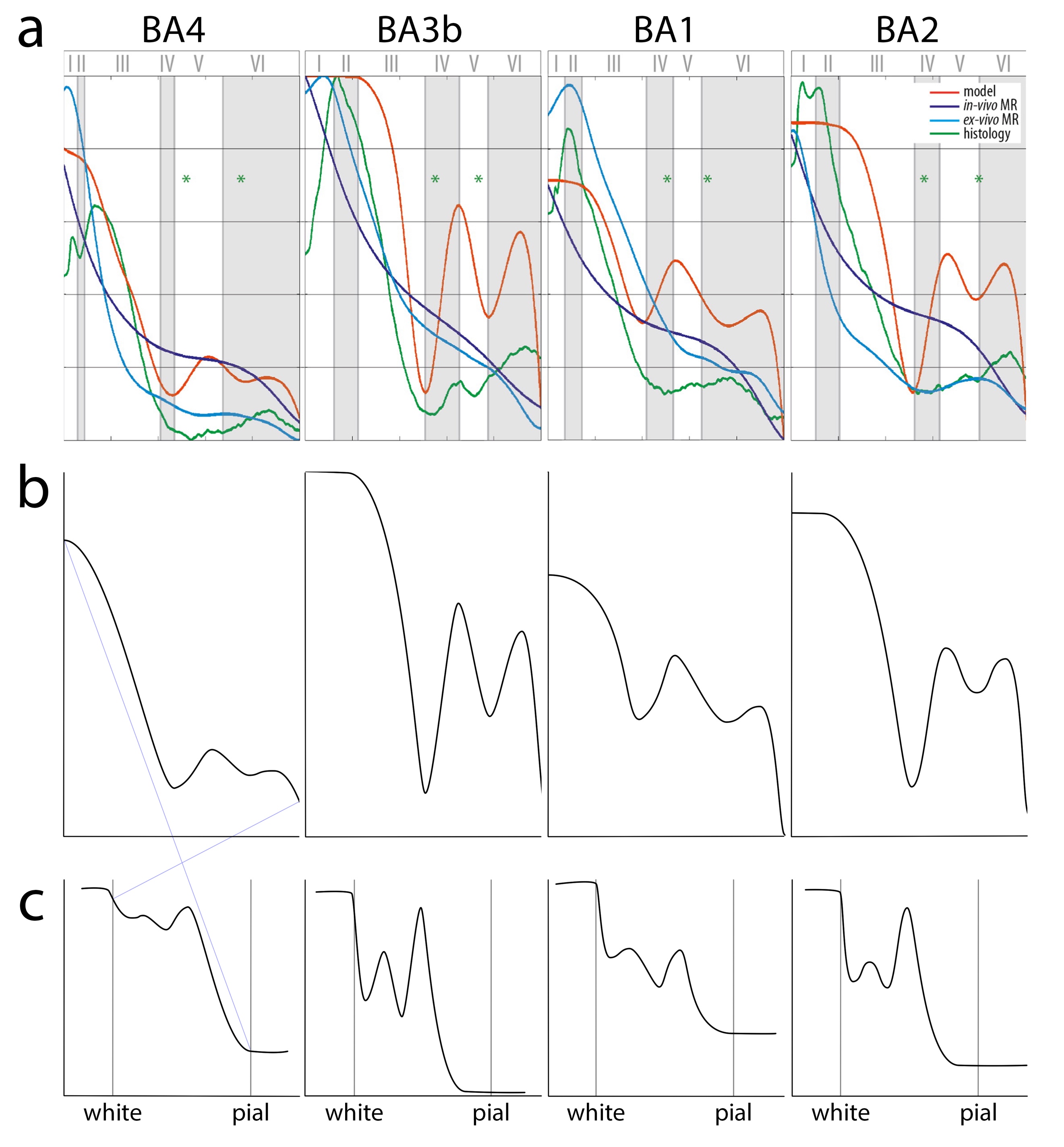


Row a) indicates models from Dinse et al (2015), where orange depicts calculated, dark blue in-vivo, light blue ex-vivo, and green histology intensity values. The calculated, or model, intensity value profiles, are copied to row b). These are mirrored in y and x to yield models in c). Profiles in c) are depicted in Figure 4 in manuscript.

Figure S3


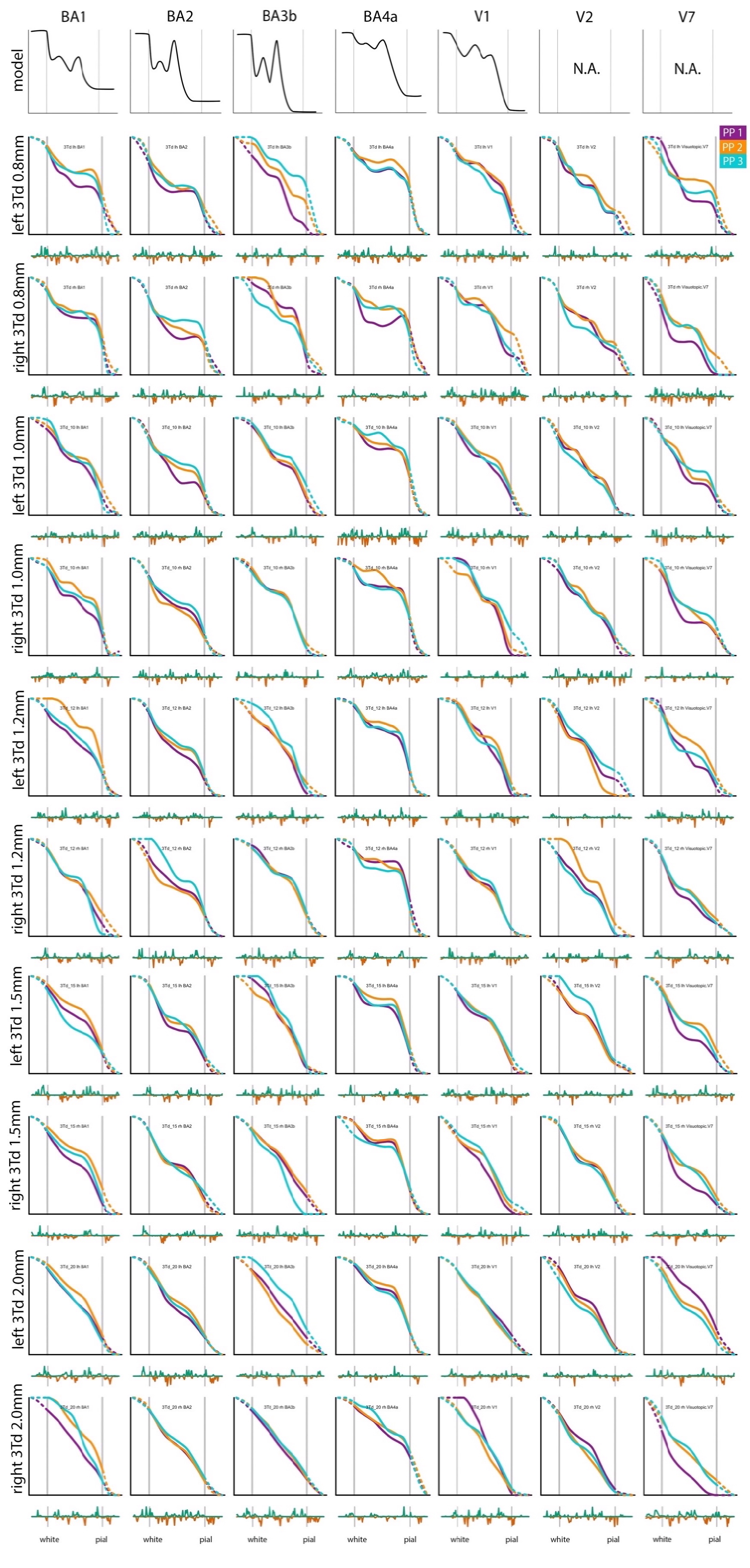


A comparison of results with different input resolutions. Top two rows contain the output of the application of the pipeline to 0.8 mm isotropic 3T T1-w volumes, where the first row contains left hemisphere and second row right hemisphere data. That volume was downsampled to yield 1.0, 1.2, 1.5 and 2.0mm isotropic volumes, that were also analysed by the pipeline. Output of that analysis is shown per two rows.

Table S1

|  |  |  | BA1 |  | BA2 |  | BA3b |  | BA4a |  | V1 |  | V2 |  | V7 |  |
| --- | --- | --- | --- | --- | --- | --- | --- | --- | --- | --- | --- | --- | --- | --- | --- | --- |
|  |  |  | lh | rh | lh | rh | lh | rh | lh | rh | lh | rh | lh | rh | lh | rh |
| PP1 | 3Td | total profiles | 768 | 559 | 1813 | 1697 | 1524 | 1560 | 1761 | 945 | 2769 | 2639 | 2954 | 4086 | 639 | 782 |
|  |  | selected profiles | 226 | 145 | 444 | 486 | 495 | 487 | 512 | 234 | 740 | 611 | 1017 | 1382 | 165 | 148 |
|  |  | % | 29 | 26 | 24 | 29 | 32 | 31 | 29 | 25 | 27 | 23 | 34 | 34 | 26 | 19 |
|  | 7Td | total profiles | 768 | 559 | 1813 | 1698 | 1526 | 1562 | 1764 | 946 | 2771 | 2639 | 2954 | 4086 | 639 | 782 |
|  |  | selected profiles | 226 | 145 | 444 | 486 | 497 | 488 | 512 | 237 | 740 | 611 | 1017 | 1382 | 165 | 148 |
|  |  | % | 29 | 26 | 24 | 29 | 33 | 31 | 29 | 25 | 27 | 23 | 34 | 34 | 26 | 19 |
| PP2 | 3Td | total profiles | 832 | 826 | 2085 | 2540 | 1443 | 1743 | 1637 | 972 | 3050 | 2933 | 3134 | 4497 | 855 | 702 |
|  |  | selected profiles | 226 | 260 | 537 | 595 | 352 | 495 | 434 | 199 | 700 | 645 | 840 | 1247 | 229 | 150 |
|  |  | % | 27 | 31 | 26 | 23 | 24 | 28 | 27 | 20 | 23 | 22 | 27 | 28 | 27 | 21 |
|  | 7Td | total profiles | 832 | 828 | 2085 | 2540 | 1443 | 1744 | 1638 | 972 | 3050 | 2934 | 3134 | 4497 | 859 | 702 |
|  |  | selected profiles | 226 | 262 | 537 | 595 | 352 | 495 | 434 | 199 | 700 | 645 | 840 | 1247 | 231 | 150 |
|  |  | % | 27 | 32 | 26 | 23 | 24 | 28 | 26 | 20 | 23 | 22 | 27 | 28 | 27 | 21 |
| PP3 | 3Td | total profiles | 657 | 704 | 1623 | 2045 | 1467 | 2038 | 1771 | 1072 | 3090 | 2883 | 3151 | 4685 | 696 | 495 |
|  |  | selected profiles | 160 | 197 | 480 | 495 | 447 | 552 | 545 | 262 | 660 | 708 | 773 | 1289 | 155 | 120 |
|  |  | % | 24 | 28 | 30 | 24 | 30 | 27 | 31 | 24 | 21 | 25 | 25 | 28 | 22 | 24 |
|  | 7Td | all profiles | 657 | 704 | 1623 | 2053 | 1468 | 2038 | 1773 | 1073 | 3090 | 2885 | 3150 | 4683 | 698 | 497 |
|  |  | selected profiles | 160 | 197 | 480 | 501 | 447 | 552 | 547 | 262 | 660 | 708 | 772 | 1291 | 157 | 122 |
|  |  | % | 24 | 28 | 30 | 24 | 30 | 27 | 31 | 24 | 21 | 25 | 25 | 28 | 22 | 25 |

Overview of total profiles, selected profiles, and percentage of selected profiles per area, hemisphere; ordered per participant and field strength.
